# Situational Analysis of Visceral Leishmaniasis in the Most Important Endemic Area of the Disease in Iran

**Published:** 2017-12-30

**Authors:** Eslam Moradi-Asl, Ahmad Ali Hanafi-Bojd, Yavar Rassi, Hassan Vatandoost, Mehdi Mohebali, Mohammad Reza Yaghoobi-Ershadi, Shahram Habibzadeh, Sadegh Hazrati, Sayena Rafizadeh

**Affiliations:** 1 Department of Medical Entomology and Vector Control, School of Public Health, Tehran University of Medical Sciences, Tehran, Iran; 2 Department of Public Health, School of Public Health, Ardabil University of Medical Sciences, Ardabil, Iran; 3 Department of Chemical Pollutants and Pesticides, Institute for Environmental Research, Tehran University of Medical Sciences, Tehran, Iran; 4 Department of Medical Parasitology and Mycology, School of Public Health, Tehran University of Medical Sciences, Tehran, Iran; 5 Department of Infection Disease, School of Medicine, Ardabil University of Medical Sciences, Ardabil, Iran; 6 Ministry of Health and Medical Education, National Institute for Medical Research Development, Tehran, Iran

**Keywords:** Visceral leishmaniasis, Seroepidemiology, GIS, Iran

## Abstract

**Background:**

Visceral leishmaniasis is one of the most important vector borne diseases in the world, transmitted by sand flies. Despite efforts to prevent the spread of the disease, cases continue worldwide. In Iran, the disease usually occurs in children under 10 years. In the absence of timely diagnosis and treatment, the mortality rate is 95–100%. The main objective of this study was to determine the spatial and temporal distribution of visceral leishmaniasis as well as its correlation with climatic factors for determining high-risk areas in an endemic focus in northwestern Iran.

**Methods:**

In this cross-sectional study, data on VL cases were collected from local health centers in Ardabil Province, Iran during 2001–2015 to establish a geodatabase using ArcGIS10.3. Data analysis was conducted using SPSS23 and ArcMap Spatial Analyst. MaxEnt model was used to determine ecologically suitable nichesfor the disease.

**Results:**

Two hotspots were found in Meshkinshahr and Germi counties with 59% and 23% of total cases, respectively. There was an increase in the incidence rate of VL in Ardabil County from 2.9 in 2009 to 9.2/100,000 population in 2015. There was no spatial autocorrelation between county and total number of cases (P> 0.05). Higher NDVI, lower altitude and southern aspects had positive effects on the presence probability of VL.

**Conclusion:**

The number of cases of this disease have been rising since 2013 and doubled in 2015. According to the derived distribution maps, the disease is spreading to new locations such as Ardabil and Namin counties.

## Introduction

Arthropod-Borne diseases are among the most important public health problems, with over one-third of the infectious diseases being transmitted by insect vectors (1). Leishmaniasis is a complex vector-borne disease caused by *Leishmania* spp (2–3). The disease, with a wide spectrum of clinical manifestations ranging from self-healing skin lesions to lethal (visceral) forms, has been reported from 101 countries in the world (4). Over 350 million people are at risk of contracting the disease (5). The most important vector of leishmaniasis in the old world is sand flies of the genus *Phlebotomus* whilst in the new world, is *Lutzomyia* spp. Clinically, leishmaniasis is divided into cutaneous leishmaniasis (CL), visceral leishmaniasis (VL) and mucocutaneous leishmaniasis (MCL) (6). Visceral leishmaniasis or kala-azar is the most lethal form of this group of diseases, and has a very high mortality rate in the absence of timely diagnosis and treatment (7). It has an annual prevalence of 0.2–0.4 million cases leading to more than 40,000 deaths (8).

Since the first report of visceral leishmaniasis in 1949 in Iran, at least four main endemic foci of the disease in Ardabil, Fars, East Azerbaijan, and Bushehr provinces have been reviewed and approved (9). Mediterranean leishmaniasis due to *L. infantum* is the most common form of disease in Iran (10), and domestic dogs and other wild canines have been identified as the main reservoirs. Although about 100–300 new cases are reported every year in Iran across the country, the main foci are in northwestern part, especially Ardabil Province (11–12). Children under 5yr old constitute over 89% of patients in the endemic areas of VL in Iran (13). The symptoms of VL include fever, hepatosplenomegaly (14) and anemia (15).

Geographic Information System (GIS) is a new technology which is now widely used in the study of diseases transmitted by arthropods (VBDs) such as the different forms of leishmaniasis. Its application has caused significant changes in data interpretation and decision-making on disease control (16). GIS technique is useful in understanding the spatial distribution of the diseases, which provides valuable information on the correlation between infection occurrences, climate and environmental variables. It is also able to identify and predict high-risk areas of the diseases. Data derived from this technique will facilitate the implementation of environmental interventions at the right time and place. There is a high correlation between the life cycle of VL and the environmental factors involved in its life cycle in terms of geographical distribution (17).

Using space technologies provides new opportunities for rapid assessment of endemic diseases, accurate and reliable estimation of the population at risk, prediction of disease distribution in areas where information is not available, and determination of appropriate strategy for the control and prevention of the disease in these areas (18). Previous studies that used GIS and RS techniques to examine the spatial distribution of VL found a correlation between the disease and environmental variables. These techniques were also used to describe host and vector ecology as well as the population at risk of contracting the disease. For example, land use was positively affected sand fly population and thus, a risk factor for VL transmission in Brazil, India and Iran (19–23).

In Iran, sand flies from genus *Phlebotomus* (*P. kandelakii*, *P. neglectus*, *P. keshishiani*, *P. perfiliewi transcaucasicus*, *P. alexandri* and *P. tobbi*) mainly transmit *L. infantum*, the causative agent of VL, from the infected canines to humans (24–31). This parasite usually infects children under the age of 10 years. Ardabil Province is the most important endemic focus of VL in Iran, and in recent years, 25–50% of visceral leishmaniasis cases have been found to occur in this province (12, 32, 33).

One of the most important serologic tests used for the diagnosis of visceral leishmaniasis is Direct Agglutination Test (DAT) (2, 34). This test has been used for the diagnosis of VL patients in Ardabil Province since 1996 (15). Like other vector-borne diseases, different environmental variables as well as demographics and human activities seem to affect the distribution and incidence of VL. These variables should thus be taken into account during disease prediction and management investigations.

The objectives of this study were to determine the geographical distribution of visceral leishmaniasis in Ardabil Province using GIS in order to identify high-risk zones in the province, and to evaluate the role of environmental and geographical variables on the disease distribution.

## Materials and Methods

### Study area

**Fig. 1.**
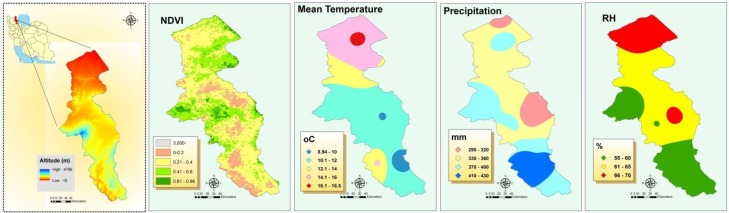
Weather and environmental situation of the Study area in Northwest of Iran

Ardabil Province is located in the northwest of Iran (37.45–39.42° N, 47.30–48.55° E). The province has an area of 17,953km^2^ and a population of 1,249,000 people, according to the last census conducted in 2011. The capital of this province is Ardabil County and according to the latest provincial demarcations, the province consists of 10 counties, 21 districts, 26 cities, 71 rural districts and 1477 permanent villages (35). Topographically, 477 villages (32.3%) are located in plain areas, 975 villages (66%) in mountain valleys, 17 villages (1.17%) in foothill areas and 8 villages (0.54%) in forested areas. Climate is variable in Ardabil Province. About 2/3 of the extent of the study area has mountainous areas and the remaining is covered by plains. Overall, the north of the province is situated at lower altitude with relatively warmer weather, whereas the central and southern regions have cool and mountain climate (Fig. 1). The area is covered by natural vegetation or agricultural fields such that the NDVI ranges between 0 and 0.86. Most common occupations of the people are agriculture, horticulture and animal husbandry. The ratio of urban to rural population in Ardabil Province is 64/36.

### Data collection and analysis

Data on VL cases were collected from Ardabil Province health centers during the last 15yr from March 2001 to the end of February 2015. The number of patients who were seropositive on DAT test at titers ≥ 1:3200 and with clinical symptoms recognized by pediatricians were recorded and registered into a geodatabase created in Excel. The geodatabase included demographic data of patients, their area of residence (county, city, rural district, village), date in which infection was diagnosed, titer of DAT test, etc. The data were then imported into ArcMap 10.3 and stored as a shapefile for mapping and statistical/spatial analysis. The impact of factors such as age, gender and residence of patients on the prevalence of VL was assessed by SPSS version 23 (Chicago, IL, USA) and chi-square analysis (CI= 95%).

Meteorological data were obtained from the Ardabil Meteorological Center during the study period. The data included annual precipitation (mm), average monthly temperature (°C), average minimum temperature (°C), average maximum temperature (°C), and relative humidity (%).

### Spatial analysis

ArcGIS 10.3 (http://www.esri.com/arcgis) was used for spatial analysis and mapping. Inverse Distance Weighted (IDW) interpolation analysis was used to prepare raster maps of the climatic variables and NDVI (Normalized difference in vegetation index) (Fig. 1), and was also used to determine high risk areas of the disease across the study area. This method assumes that the variable being mapped decreases in influence with distance from its sampled location. The “extract values to points” tool in spatial analyst was used to extract the cell values of the prepared raster layers to the VL positive places.

Spatial autocorrelation of VL cases in the different counties of the study area was estimated. The spatial autocorrelation tool in ArcGIS measures spatial autocorrelation based on both feature locations and feature values simultaneously. Given our VL cases data and the associated attribute (county border), we evaluated the pattern of the disease (clustered, dispersed, or random). Moran’s I Index value was calculated, and as well, both a z-score and p-value (P< 0.05) were also calculated and were used to evaluate the significance of the index (36).

This index is given as:

$$I=\frac{n}{S0}\frac{\sum_{\text{i}=1}^{\text{n}}.\sum_{i=0}^{n}wi,jzizj}{\sum_{i=1}^{n}z_{i}^{2}}$$

where z_i_ is the deviation of an attribute for feature *i* from its mean (xi-X), wi, j, is the spatial weight between feature i and j, n is equal to the total number of features, and S0 is the aggregate of all the spatial weights: $S0=\sum_{i=1}^{n}\sum_{j=1}^{n}wi,j$

The z_I_- statistic score is calculated as: $z_{\text{I}}=\frac{I-E\left[I\right]}{\surd V\left[I\right]}$ where: E[*I*]= −1/(n−1), V[*I*]= E[*I*^2^]-E[*I*]^2^

### Modeling VL distribution

Maximum Entropy (MaxEnt) model ver 3.3.3 (37, 38) was used for this purpose. We used coordinates with 3 or more cases of VL as presence points of the disease where *L. infantum* is circulating among sand fly, canine reservoirs and humans. 19 bioclimatic variables (Table 1) as well as altitude layer were downloaded from the worldclim database with a spatial resolution of 1km^2^ (version1.4, http://www.worldclim.org/bioclim). Using ArcMap 10.3 and surface analysis slope and aspect (slope direction) layers were derived. Normalized difference vegetation index (NDVI) was obtained from MODIS images at the same spatial resolution. The contribution of environmental variables and bioclimatic variables were tested by Jackknife analysis. All variables with no contribution (0 values) based on the test results were excluded from the final analysis. Eighty percent of occurrence records were selected for training model and the remaining 20% for model testing.

**Table 1.** Variables used for MaxEnt modeling of VL distribution in Ardabil Province, Northwest of Iran

**Variable**	**Description**	**Contribution (%)**
**Bio1**	Annual mean temperature (°C)	0
**Bio2**	Mean diurnal range: mean of monthly (max temp–min temp) (°C)	2.8
**Bio3**	Isothermality: (Bio2/Bio7)× 100	16.4
**Bio4**	Temperature seasonality (SD× 100)	17.3
**Bio5**	Maximum temperature of warmest month (°C)	0
**Bio6**	Minimum temperature of coldest month (°C)	0
**Bio7**	Temperature annual range (Bio5–Bio6) (°C)	0.1
**Bio8**	Mean temperature of wettest quarter (°C)	0.4
**Bio9**	Mean temperature of driest quarter (°C)	0
**Bio10**	Mean temperature of warmest quarter (°C)	0
**Bio11**	Mean temperature of coldest quarter (°C)	0.5
**Bio12**	Annual precipitation (mm)	0
**Bio13**	Precipitation of wettest month (mm)	0
**Bio14**	Precipitation of driest month (mm)	0
**Bio15**	Precipitation seasonality (coefficient of variation)	5.9
**Bio16**	Precipitation of wettest quarter (mm)	0.1
**Bio17**	Precipitation of driest quarter (mm)	1.6
**Bio18**	Precipitation of warmest quarter (mm)	1.2
**Bio19**	Precipitation of coldest quarter (mm)	2.3
**Altitude**	Elevation from the sea level (m)	0
**Slope**	Slope of the area (%)	1.4
**Aspect**	direction of slope (Degree)	15.5
**NDVI**	−1 to +1	34.6

## Results

### Demography and the disease

**Fig 2.**
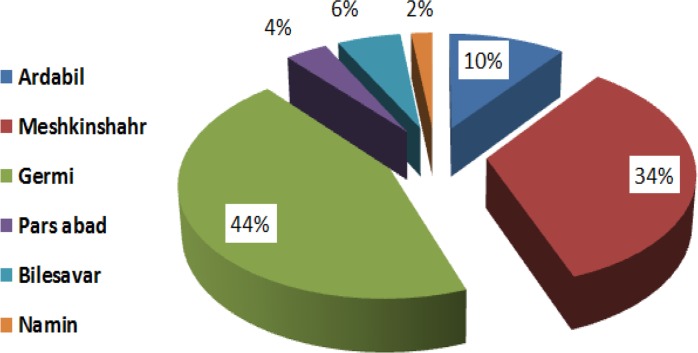
Percent of VL infected villages in different counties of Ardabil Province, Northwest of Iran, 2001–2015

**Fig. 3.**
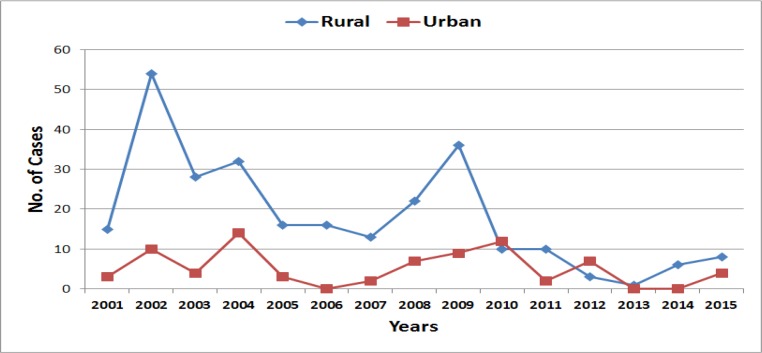
Cases of Visceral Leishmaniasis in urban/rural areas of Ardabil Province of Iran, 2001–2015

Temporal distribution of the disease in the province shows that the highest prevalence occurred in March. In other words, the seasonal disease outbreak was higher in spring (32%) and winter (31.5%), whilst the minimum number of total cases was reported in autumn (17.6%), (Fig. 2). Most of the cases of visceral leishmaniasis were recorded in 2001, 2002 and 2004, and the least one in 2013 and 2014. However, the trend was increasing from 2013 (Fig. 3).

Based on age group distribution of the disease, more than 86% of the patients were children under 4yr, of whom 44.67% were younger than 2 years. Two percent of the patients were over 10yr (Table 2). According to gender distribution, 58.8% of the patients were male and remaining female (Table 3). The highest DAT titer (1: 3200) was recorded in 32.27% of the cases, and sera from 6% of the patients demonstrated the lowest DAT titer at 1:25600 (Table 3).

**Table 2.** Frequency of VL by age groups in Ardabil Province of Iran, 2001–2015

**County**	**Ardabil**	**Bilahsavar**	**Germi**	**Meshkinshahr**	**Namin**	**Parsabad**	**Khalkhal**	**Nir**	**Kowsar**	**Sareyn**	**Total**
**Age**	No. (%)	No. (%)	No. (%)	No. (%)	No. (%)	No. (%)	No. (%)	No. (%)	No. (%)	No. (%)	No. (%)
**<2**	13(40.62)	6(46.15)	46(57.5)	85(41.46)	0	5(38.46)	0	0	0	0	155(44.67)
**2–4**	18(56.25)	6(46.15)	19(23.75)	90(43.9)	4(100)	8(61.54)	0	0	0	0	145(41.78)
**5–7**	1(3.15)	0	7(8.75)	23(11.22)	0	0	0	0	0	0	31(8.93)
**8–10**	0	0	3(3.75)	6(2.93)	0	0	0	0	0	0	9(2.60)
**>10**	0	1(7.70)	5(6.25)	1(0.49)	0	0	0	0	0	0	7(2.02)
**Total**	32(100)	13(100)	80(100)	205(100)	4(100)	13(100)	0	0	0	0	347(100)

**Table 3.** Number of visceral leishmaniasis cases in Ardabil Province of Iran by gender, 2001–2015

**DAT**	**1:3200**		**1:6400**		**1:12800**		**1:25600**		**1:51200**		**1:102400**		**Total**	

**County**	**Male**	**Female**	**Male**	**Female**	**Male**	**Female**	**Male**	**Female**	**Male**	**Female**	**Male**	**Female**	**Male**	**Female**
**Ardabil**	7	4	4	3	2	4	1	0	0	2	4	1	18	14
**Bilahsavar**	2	2	2	1	1	0	1	0	1	1	2	0	9	4
**Germi**	21	20	10	3	7	2	4	4	1	3	5	0	48	32
**Meshkinshahr**	24	25	21	12	18	16	9	2	15	9	33	21	120	85
**Namin**	0	2	2	0	0	0	0	0	0	0	0	0	2	2
**Parsabad**	4	1	2	2	0	0	0	0	0	2	1	1	7	6
**Khalkhal**	0	0	0	0	0	0	0	0	0	0	0	0	0	0
**Nir**	0	0	0	0	0	0	0	0	0	0	0	0	0	0
**Kowsar**	0	0	0	0	0	0	0	0	0	0	0	0	0	0
**Sareyn**	0	0	0	0	0	0	0	0	0	0	0	0	0	0
**Total**	58	54	41	21	28	22	15	6	17	17	45	23	204	143

**Fig. 4.**
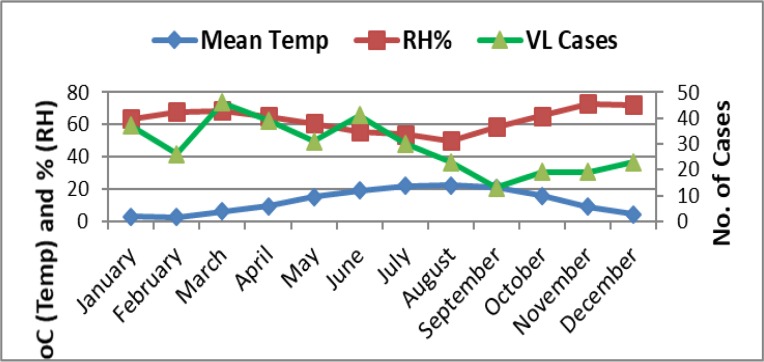
The monthly incidence of Visceral Leishmaniasis in Ardabil Province of Iran and weather data, 2001–2015

A total of 347 positive cases of Kala-Azar have been recorded in the past 15yr across the province, the maximum number of cases were recorded in Meshkinshahr (59.1%) and Germi (23.1%), and the minimum number of cases were occurred in Namin (1.2%) (Fig. 3). The distribution map of the disease shows that five out of the 10 counties in Ardabil Province had local cases, which shows that the highest number of cases occurred in the central parts of the province (Meshkinshahr and Germi) and their adjacent counties (Fig. 4).

Overall, 93 villages (6.3%), 35 rural districts (49.3%), 14 districts (66.66%) and 10 counties (38.5%) had reported cases of visceral leishmaniasis. Most of the infected villages (74%) were located in mountain valleys, whereas 19.24%, 4.8% and 1.93% of the villages were located in the plains, foothills and forest areas, respectively. Based on the number of cases per area, 85 areas (77.4%) had less than 5 cases, 12 areas (12.9%) reported 5–10 cases, 4 areas (4.3%) had 10–15 cases, and 4 areas (5.4%) recorded more than 15 VL cases. The results of this study show that severity and frequency of VL was higher in Meshkinshahr, but in terms of spatial distribution, 41% of the villages in Germi County with a population of 84,267 peoples were found to have the highest number of cases (Fig. 2). Most cases occurred in rural areas (Fig. 3). The cumulative monthly reported cases of VL in the study area show that it was more prevalent during the first half of the year, but dropped from June to September, and then increased again in the latter part of the year (Fig. 4). The occurrence of VL in the study area had a significant correlation with the mean temperature (P< 0.001) and mean relative humidity (P< 0.000) in the different months.

### Spatial analysis

**Fig. 5.**
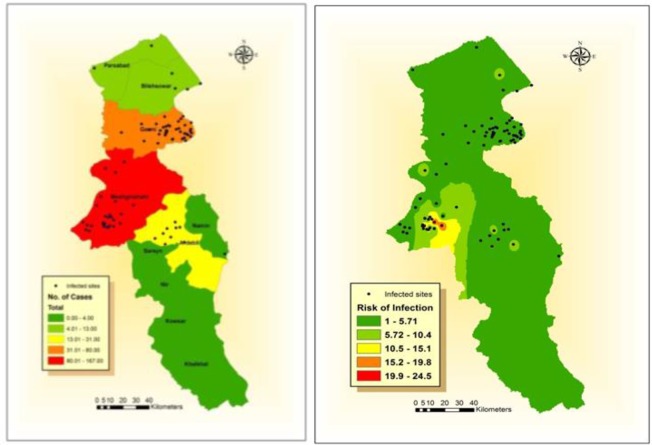
Distribution map (left) and IDW interpolation (right) of visceral leishmaniasis in different counties of Ardabil Province of Iran including infected sites, 2001–2015

**Fig. 6.**
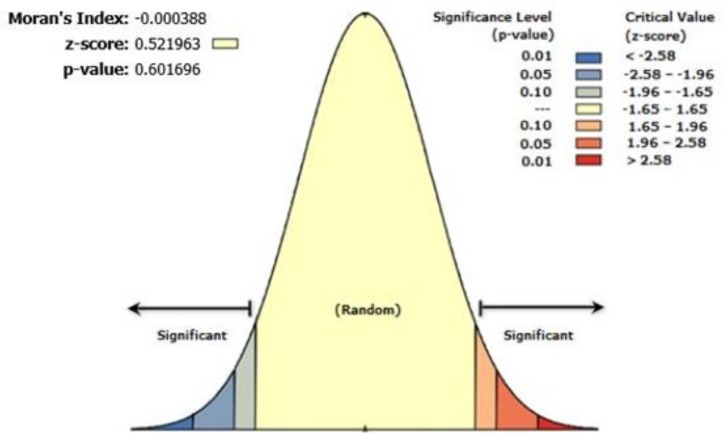
Moran’s I autocorrelation analysis of visceral leishmaniasis cases in different counties of Ardabil Province of Iran, 2011–2015

Spatial distribution of the infection sites and ranking of the counties according to the incidence of VL revealed that most of the disease cases were reported in Meshkinshahr County followed by Germi, whilst 4 counties had no reported cases of VL during the study period (Fig. 5). Interpolation of the disease infection showed Meshkinshahr areas as the hot spot of the disease (Fig. 5). Moran spatial autocorrelation analysis showed that there was no spatial correlation between the different counties in terms of the total number of cases recorded during the study period, and the distribution was random (Z-Score: 0.521963, P> 0.05). Results of the interpolation analysis also revealed the central regions of the province as hot spots of VL (Fig. 6). According to the climatic and environmental data, VL infections were high in areas with the following climatic/environmental variables: at an altitude between 58–1935m above sea level, relative humidity between 56.33–70.32%, total annual precipitation between 288–382mm, minimum temperature between −4.42–5.99 °C, maximum temperature between 21.52–28.49 °C, average temperature between 9.86–15.54 °C, and NDVI between 0.141–0.749.

### Modeling the ecologically suitable areas of VL

**Fig. 7.**
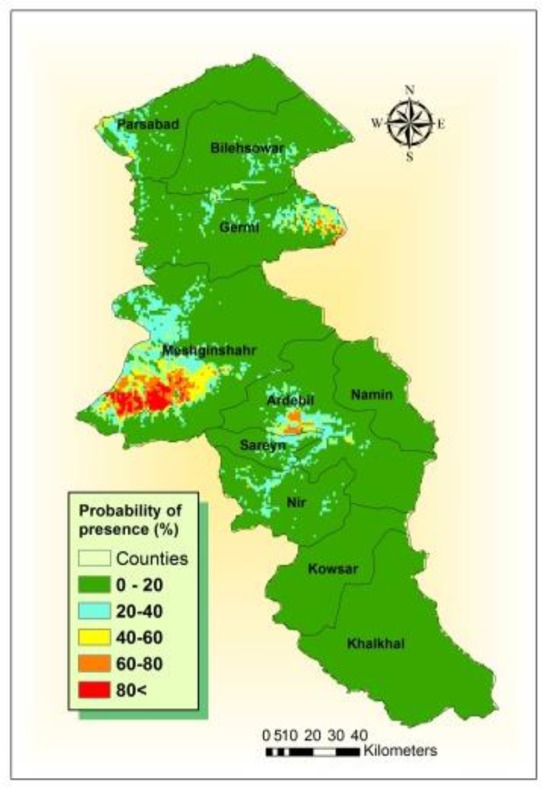
Ecologically suitable areas for VL occurrence in Ardabil Province, Northwest of Iran

**Fig. 8.**
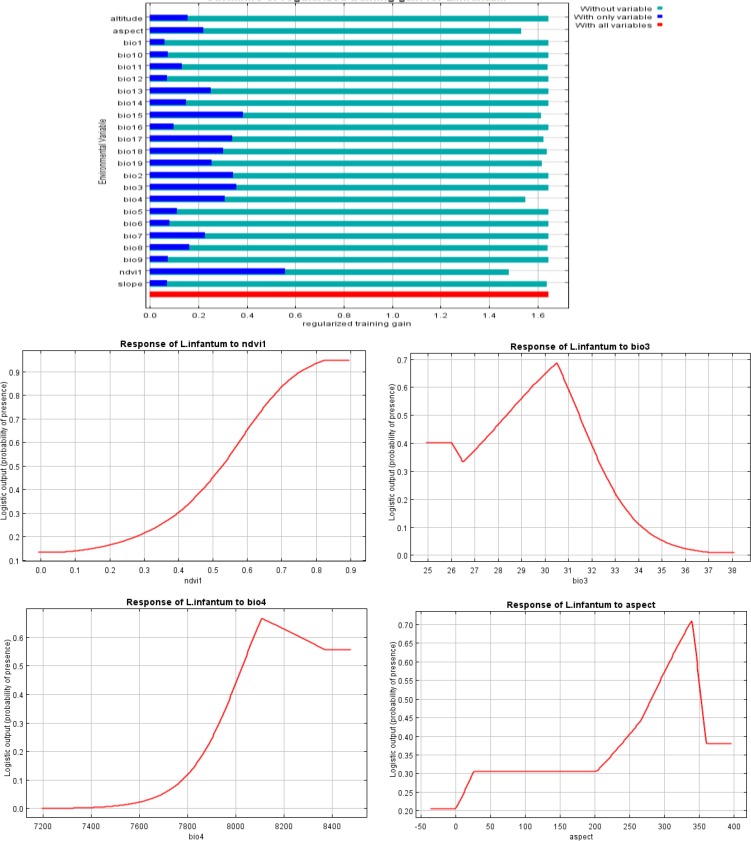
Result of jackknife test of variables importance for VL in Ardabil Province, northwest of Iran

Results of the MaxEnt model showed a large extent of the province with presence probability less than 20%, and the most ecologically suitable areas of VL occurrence were identified in three hotspots (Fig. 7) in Meshkinshahr, Germi and Ardabil counties with a population of 799,788 at risk. The area under receiver operating characteristic (ROC) curve (AUC) was 0.945 and 0.885 for training and test data, respectively. According to the jackknife test, the environmental variable with highest gain when used in isolation was NDVI (Table 1), such that higher NDVI values had positive effect on the presence probability of VL. Bio4, Bio3 and aspect were the other environmental variables with highest contribution to the model (Fig. 8). Considering altitude, the model we used found a positive trend to about 1250m above sea level, but higher altitudes demonstrated a negative presence probability for the disease.

## Discussion

The results of the present study showed that VL has expanded across the study area over last 15 years, with cases of the disease reported in half of the counties in the province. From 1984 to 1989, Meshkinshahr was the most infected area in the province (39). Other studies have also reported the occurrence of VL in Meshkinshahr, Germi, Bilehsavar, Parsabad and Ardabil counties (40). In line with the results of these studies, our findings confirmed Meshkinshahr as the most infected county, and the spatial expansion of the disease to other counties in recent years were also observed. Severity and frequency of VL was high in Meshkinshahr County whilst the distribution of VL was higher in Germi County compared with the other areas. In this study, males were found to be more infected.

Studies conducted in other countries showed a decreasing trend in the disease incidence and an increasing trend in the spatial distribution of the disease (41–44). Although the results of the above studies, conducted in India and Brazil, are consistent with our results, in Afghanistan, there has been 300% increase in VL cases due to war and displacement of the inhabitants (45).

Based on age distribution analysis, 98% of patients were children under 10yr, of whom more than 70% were less than 2yr old. Indicating the age of infection has decreased over the past years (P< 0.05). This result is inconsistent with the results of a study conducted on the disease in this area during 1986–2005 (46) and the findings of Moradi-Asl et al. (13) in Meshkinshahr endemic focus of VL, as well as with a study conducted in Italy (47). In Brazil, 37% of the cases occurred in patients less than 15yr old whereas only 13% of the cases occurred among patients under 1 year (48). The difference between our study and the studies mentioned above is due to the difference between the parasitic agents circulating in the different study areas. In the Mediterranean basin, *L. infantum* is the main causative agent of VL, but *L. donovani* is the main cause of VL in other parts of the world (9).

There was no significant difference between males and females (P> 0.05) in terms of the incidence of the disease. In Iran and Pakistan, sex ratio of VL patients was 2:1 (M: F) (49–50). Males were infected 1.1 times more than females in Brazil (51), but another study reported a sex ratio similar to that of our study (48).

Seasonal studies of VL infections showed that over 63% of the reported cases in our study occurred in spring and winter, but the incidence was low in summer and autumn (P> 0.05). Although a decreasing (P< 0.001) trend was generally observed, occurrence of the disease exhibited a sinusoidal pattern during every 2–3 years. Spatial distribution of the disease shows that it has expanded to new areas in the province including Ardabil and Namin counties. To prevent probable epidemics, it is recommended that a comprehensive study on the infection of vectors and reservoir hosts in the area be conducted in order to determine areas with epidemic potential. This can be done by modeling studies. These types of research have been conducted in some parts of Iran and other countries (31, 41, 52).

Most of the cases of VL were recorded in the central and western rural districts of Meshkinshahr, Muran, central rural districts of Germi, and central rural districts of Ardabil, Bilehsavar and Parsabad counties. In these areas, marginalization is higher than in other counties; animal husbandry and farming are the main occupations of the people, and VL reservoir (domestic and wild canines) population is higher than in the urban areas. Although VL cases were different between the counties, Moran’s spatial statistical analysis showed that the pattern of the disease distribution was random.

Different sand fly species which are vectors of VL are distributed across the country (24–26, 53–54), and among them, three species (*P. kandelakii*, *P. perfiliewi* and *P. tobbi*) exist in the present study area. Presence of seropositive reservoirs has also been reported in the province (11, 55–60). Modeling the distribution of vectors and reservoirs and overlaying the outputs of the model will thus be useful in identifying the potential hot spots. In India, the transmission rate of VL was higher in areas with high *P. argentipes* density, and areas with high VL incidence (serologically) in reservoir population, had more positive human cases in Western Europe (62).

Contrary to the findings in Brazil (20) and north of India (18), we found that the disease is more prevalent in areas where NDVI is higher. This is due to the difference between the ecological needs of both the parasite and vectors of VL in the different study areas. On the other hand, in our study area, *L. infantum* is the main cause of the disease which is transmitted by *P. kandelakii*, *P. perfiliewi* and *P. tobbi* (31). However, in India, *L. donovani* is transmitted by *P. argentipes* which prefers lowlands with higher temperatures and lower NDVI. In Meshkinshahr County, the number of freezing days, rainfall and humidity were more effective VL risk predictors (21). We found negative presence probability for the disease at altitudes higher than 1250m above sea level. This is in accordance with the findings of other studies (18, 21, 23). Higher altitudes have lower temperatures that may prevent the cycle of VL transmission leading to failure to meet ecological requirements, especially the optimum temperature for both parasite and vector(s). Similar to our findings, Ghatee et al. (22) reported that temperature is the most effective variable that affects the distribution of VL.

Like other vector-borne diseases, increased exposure to the vectors leads to a higher risk of VL infections. Nomads living in the study area are mainly livestock farmers who rear sheepdogs, and mostly live outdoors during VL transmission seasons. The chance of sand fly bites is thus higher among the people in this area. This is also a major risk factor for the distribution of the disease, especially to the new areas such as Ardabil and Namin counties, where VL was not previously reported. Previous studies conducted in Iran confirmed the role of travel and nomadic life style on the incidence of visceral leishmaniasis (22–23).

## Conclusions

Despite the efforts for control and preventing VL, many new cases of VL in humans and reservoirs (dogs) continue to occur in Ardabil Province, which is an old endemic focus of VL in Iran. Even though there is a decline in the total number of cases, the disease continues to spread to new areas and among the reservoirs, especially in domestic dogs. This should be considered in planning preventive measures to keep the disease under control. We suggest that a comprehensive program for monitoring and surveillance of the disease in humans and reservoirs should be implemented using GIS, and as well, climate change and its effects on the disease should be considered.
